# A Peptidoglycan Amidase Mutant of *Burkholderia insecticola* Adapts an L-form-like Shape in the Gut Symbiotic Organ of the Bean Bug *Riptortus pedestris*

**DOI:** 10.1264/jsme2.ME20107

**Published:** 2020-11-11

**Authors:** Shiori Goto, Tsubasa Ohbayashi, Kazutaka Takeshita, Teruo Sone, Yu Matsuura, Peter Mergaert, Yoshitomo Kikuchi

**Affiliations:** 1 Graduate School of Agriculture, Hokkaido University, 060–8589 Sapporo, Japan; 2 Université Paris-Saclay, CEA, CNRS, Institute for Integrative Biology of the Cell (I2BC), 91198, Gif-sur-Yvette, France; 3 Institute for Agro-Environmental Sciences, National Agriculture and Food Research Organization (NARO), 305–8604 Tsukuba, Japan; 4 Faculty of Bioresource Sciences, Akita Prefectural University, 010–0195 Akita, Japan; 5 Research Faculty of Agriculture, Hokkaido University, 060–8589 Sapporo, Japan; 6 Tropical Biosphere Research Center, University of the Ryukyus, 903–0213 Okinawa, Japan; 7 Bioproduction Research Institute, National Institute of Advanced Industrial Science and Technology (AIST), Hokkaido Center, 062–8517 Sapporo, Japan

**Keywords:** *Burkholderia*, gut symbiosis, cell shape, *amiC*, L-form

## Abstract

Bacterial cell shapes may be altered by the cell cycle, nutrient availability, environmental stress, and interactions with other organisms. The bean bug *Riptortus pedestris* possesses a symbiotic bacterium, *Burkholderia insecticola*, in its midgut crypts. This symbiont is a typical rod-shaped bacterium under *in vitro* culture conditions, but changes to a spherical shape inside the gut symbiotic organ of the host insect, suggesting the induction of morphological alterations in *B. insecticola* by host factors. The present study revealed that a deletion mutant of a peptidoglycan amidase gene (*amiC*), showing a filamentous chain form *in vitro*, adapted a swollen L-form-like cell shape in midgut crypts. Spatiotemporal observations of the Δ*amiC* mutant in midgut crypts revealed the induction of swollen cells, particularly prior to the molting of insects. To elucidate the mechanisms underlying *in vivo*-specific morphological alterations, the symbiont was cultured under 13 different conditions and its cell shape was examined. Swollen cells, similar to symbiont cells in midgut crypts, were induced when the mutant was treated with fosfomycin, an inhibitor of peptidoglycan precursor biosynthesis. Collectively, these results strongly suggest that the *Burkholderia* symbiont in midgut crypts is under the control of the host insect via a cell wall-attacking agent.

Ever since Antonie van Leeuwenhoek observed the amazing diversity of bacteria and bacterial shapes for the first time in the 17^th^ century using his self-made microscope, bacterial cell morphology has attracted the attention of a broad range of microbiologists. Bacterial cell shape is intrinsically controlled by the cell wall, which is composed of the peptidoglycan sacculus, and by cytoskeleton protein complexes, such as the tubulin homolog FtsZ and actin-like MreB, which function during cell division and elongation (Egan *et al.*, 2020). Under specific conditions, such as the inhibition of bacterial cell wall synthesis by antibiotics or lysozyme, the cell shape may change to a spherical form called the “L-form”. L-form bacteria proliferate without a cell wall or with only remnants of it, and their spherical cell shape represents the energetically most stable form for adaptation to cell turgor (Strang *et al.*, 1991; Schmidtke and Carson, 1999; Allan *et al.*, 2009).

Bacterial morphology is also influenced by internal factors, such as the bacterial growth phase, and by external factors, including nutrient availability, environmental and chemical stresses, physical constraints, predation, and life inside other organisms (Young, 2006; van Teeseling *et al.*, 2017). Bacteria that colonize environments inside other organisms, such as endosymbionts or pathogens, sometimes adapt forms that markedly deviate from their regular shape in the free-living state. A well-known example of this cell shape alteration is in the nitrogen-fixing legume-rhizobium symbiosis when rhizobial symbionts colonize the symbiotic organ, the root nodule, developed by the host plant. Rhizobia are typical rod-shaped *alpha-* or *beta-Proteobacteria* in the free-living state in soil and under *in vitro* culture conditions (Oke and Long, 1999). The symbiotic bacteria that colonize the plant cells of the root nodule differentiate into a specialized nitrogen-fixing form called a “bacteroid”. In some legumes, bacterial cell division is inhibited during the formation of bacteroids, whereas cell growth and genome replication continues, resulting in polyploid, extremely enlarged bacterial cells that may be elongated, branched, or spherical (Oke and Long, 1999; Mergaert *et al.*, 2006; Czernic *et al.*, 2015). Defensin-like membrane-attacking antimicrobial peptides called “Nodule-specific Cysteine Rich peptides (NCRs)” are strongly expressed in root nodules (Mergaert *et al.*, 2003), and induce morphological and physiological alterations in rhizobium bacteroids (Mergaert, 2018; 2020).

Intracellular symbionts are also common in insects, which carry them in specific organs called bacteriomes composed of symbiont-infected cells or bacteriocytes (Baumann, 2005). Bacteriocyte-associated endosymbionts are generally unable to grow in a free-living state outside of their insect host, but have free-living close relatives in *alpha-*, *beta-*, and *gamma-Proteobacteria* or *Bacteroidetes*. These intracellular insect symbionts are often large and have markedly different morphologies from their rod-shaped free-living relatives. Similar to the rhizobium bacteroids in legumes, bacteriocyte symbionts may be spherical or elongated and sometimes enlarged to extreme sizes. Previous studies reported that these symbionts have very unusual, irregular, and pleomorphic shapes, forming balloon structures with invaginations or even rosette-like forms (Login *et al.*, 2011; Shigenobu and Wilson, 2011; Hirota *et al.*, 2017; Lukasik *et al.*, 2017; Okude *et al.*, 2017; Bublitz *et al.*, 2019). These differentiated bacterial forms of insect and plant endosymbionts may adapt space-filling shapes to optimally occupy the greatest volume available in their host cells.

The bean bug *Riptortus pedestris* possesses a gut symbiotic bacterium, *Burkholderia insecticola*, in the crypt-bearing posterior region of the midgut (the 4^th^ section of the midgut, M4), which harbors more than 100 million symbiont cells (Takeshita and Kikuchi, 2017; Kaltenpoth and Flórez, 2020; Ohbayashi *et al.*, 2020). Eggs and hatchlings of *R. pedestris* are aposymbiotic (*i.e.* symbiont-free) and the insect acquires *B. insecticola* specifically from ambient soil during its development (Kikuchi *et al.*, 2007; 2011). A bacteria-sorting organ of the midgut, called the “constricted region”, is crucial for the acquisition of symbionts because it separates the anterior digestive regions of the midgut from the posterior symbiotic compartment (Ohbayashi *et al.*, 2015). The flagellar motility of *B. insecticola* (Ohbayashi *et al.*, 2015) in addition to other as yet unknown molecular features (Itoh *et al.*, 2019) are pivotal for passing through the constricted region and reaching the crypt-bearing symbiotic gut region. After colonization, morphological and physiological alterations occur in *B. insecticola* (Ohbayashi *et al.*, 2019); the cell shape becomes spherical, the DNA content per cell decreases, and the lipopolysaccharide (LPS) of the cell envelope is partially altered. At the transcriptomic level, several metabolic pathways, including assimilation pathways of host metabolic waste, such as sulfate and allantoin, are specifically up-regulated *in vivo*, indicating that *B. insecticola* proliferates in the bean bug midgut by recycling the metabolic waste of the host (Ohbayashi *et al.*, 2019). However, the underlying mechanisms and molecules of the host midgut that induce morphological and physiological alterations in the symbiont currently remain unknown.

Bacterial N-acetylmuramyl-L-alanine amidase encoded by *amiC* is involved in septal peptidoglycan cleavage during cell division (Heidrich *et al.*, 2001). An *amiC* (BRPE64_ACDS22630) deletion mutant of *B. insecticola* (Δ*amiC*) has a filamentous form composed of chains of unseparated cells because cell division is incomplete. Consequently, the mutant has less motility and infection ability (Lee *et al.*, 2015). The chain form of the Δ*amiC* mutant is nutrient-dependent; although the mutant forms chains in nutrient-rich YG (yeast extract and glucose) medium, it forms separate cells in minimal medium and its motility and infection ability are restored (Lee *et al.*, 2015).

In the present study, we revealed that morphological alterations were more prominent in the Δ*amiC* mutant inside the insect midgut, in which the mutant becomes spherical and enlarged, than under *in vitro* conditions. Furthermore, to clarify the mechanisms underlying morphological changes in the symbiont inside the midgut crypts, we examined the effects of nutrients, stress agents, and antibiotics on the cell morphologies of the symbiont, and found that the antibiotic fosfomycin mimicked the swollen shape *in vitro*.

## Materials and Methods

### Insects and bacterial strains

The bean bug *R. pedestris* TKS1 inbred line is derived from a pair of wild insects collected from a soybean field in Tsukuba, Ibaraki, Japan in 2007 and has been maintained in the laboratory for more than ten years. Insects were reared in a container at 25°C under a long-day regimen (16 h light, 8 h dark) and fed dry soybean seeds and a cotton pad containing distilled water with 0.05% ascorbic acid. The container was replaced twice a week. In infection experiments, newborn insects were placed in a Petri dish and fed as described above.

The GFP-labeled *B. insecticola* wild-type strain RPE225 (Kikuchi and Fukatsu, 2014) and GFP-labeled Δ*amiC* mutant (Lee *et al.*, 2015) were used in the present study. A Tn7-GFP mini-transposon was used to generate a fluorescent-labeled bacterial strain, as described previously (Kikuchi and Fukatsu, 2014). These bacteria were cultured at 27°C with agitation at 150 rpm in YG medium (yeast extract 5.0‍ ‍g L^–1^, glucose 4.0‍ ‍g L^–1^, and NaCl 1.0‍ ‍g‍ ‍L^–1^) or in MMGlc medium (minimum medium with glucose as the sole carbon source) (KH[PO_4_]_2_ 78‍ ‍mM, KH_2_PO_4_ 122‍ ‍mM, [NH_4_]_2_SO_4_ 7.57‍ ‍mM, NaCl 3.42‍ ‍mM, MgSO_4_·7H_2_O 0.405‍ ‍μM, FeSO_4_·7H_2_O 8.85‍ ‍μM, EDTA·2Na 11.3‍ ‍μM, and glucose 1.0‍ ‍g L^–1^) supplemented with rifampicin 10‍ ‍μg mL^–1^ and kanamycin 30‍ ‍μg‍ ‍mL^–1^. Bacto agar 15.0‍ ‍g L^–1^ was added for solid media.

### Oral administration of symbionts to the bean bug

Three days after hatching, water was removed from the plastic dish containing newly molted 2nd instar nymphs and they were kept overnight without water. This water deprivation stimulated the instant drinking of a bacterial suspension the next day, resulting in the efficient establishment of symbiotic infection. The *B. insecticola* wild type and Δ*amiC* mutant were pre-cultured in 3‍ ‍mL MMGlc medium containing 30‍ ‍μg mL^–1^ kanamycin at 27°C and 150 rpm in a rotary incubator; 200‍ ‍μL of the overnight culture was inoculated into 3‍ ‍mL MMGlc and incubated at 27°C and 150 rpm until the exponential growth phase. After the confirmation of bacterial motility by microscopic observations, bacterial density was adjusted to 10^7^‍ ‍cells‍ ‍mL^–1^ by measuring optical density, and the bacterial suspension was provided to insects as their drinking water. These insects were maintained until dissection and further analyses.

### Quantitative PCR

To assess the number of *B. insecticola* symbiont cells colonizing M4 crypts, DNA extraction was performed from dissected M4 crypts infected with the wild type or Δ*amiC* mutant using the QIAmp DNA Mini kit (Qiagen). A 150-base pair fragment of the *dnaA* gene was amplified by real-time quantitative PCR using KAPA SYBR Fast qPCR polymerase (KAPA Biosystems) and the primer set BSdnaA-F (5′-AGC GCG AGA TCA GAC GGT CGT CGA T-3′) and BSdnaA-R (5′-TCC GGC AAG TCG CGC ACG CA-3′) (Kikuchi and Fukatsu, 2014). The PCR temperature profile was set to 95°C for 3‍ ‍min, 40 cycles of 95°C for 3‍ ‍s, 55°C for 20‍ ‍s and 72°C for 15‍ ‍s, and then 95°C for 5‍ ‍s, 65°C for 1‍ ‍min, and 97°C for 30‍ ‍s using the LightCycler^®^ 480 Real-Time PCR System (Roche Life Science). The number of *B. insecticola* symbiont cells was calculated based on a standard curve for the *dnaA* gene with 10, 10^2^, 10^3^, 10^4^, 10^5^, 10^6^, and 10^7^ copies per reaction of the target PCR fragment.

### *In vitro* induction of swollen cells

The *B. insecticola* wild type and Δ*amiC* mutant were pre-cultured in MMGlc medium, with 30‍ ‍μg mL^–1^ kanamycin for the mutant, at 30°C and 150 rpm overnight. The overnighter was diluted with fresh MMGlc medium and incubated until the exponential phase. Bacterial cells were collected by centrifugation at 15,000 rpm at room temperature for 5‍ ‍min. The bacterial pellet was washed with MMnoC medium (minimum medium without any carbon source). Bacterial density was adjusted to OD_600_=0.05 by MMnoC medium supplemented with a carbon source: glucose 0.5%, fructose 0.5%, mannitol 0.5%, yeast extract 0.3%, or maleic acid 0.2%. In the case of the culture with MMnoC medium without any carbon source, bacterial density was adjusted to OD_600_=0.5, and these bacteria were then incubated at 27°C with agitation at 150 rpm. Regarding stress exposure conditions, after washing with MMGlc medium, bacterial density was adjusted to OD=0.05 using MMGlc medium. Stress compounds were added as follows: lysozyme from chicken egg white (c-type lysozyme; Sigma) 2.0‍ ‍mg mL^–1^, polymyxin B (Sigma) 25‍ ‍μg mL^–1^, H_2_O_2_ 125‍ ‍μM, or sodium dodecyl sulfate (SDS; Sigma) 125‍ ‍μM. In the case of exposure to fosfomycin (FOS; Sigma) 50‍ ‍μg mL^–1^, bacterial density was adjusted to OD_600_=0.05 in MMMSM (minimum medium with magnesium-sucrose-maleic acid) medium (KH[PO_4_]_2_ 78‍ ‍mM, KH_2_PO_4_ 122‍ ‍mM, [NH_4_]_2_SO_4_ 7.57‍ ‍mM, NaCl 3.42‍ ‍mM, MgSO_4_·7H_2_O 0.405‍ ‍μM, FeSO_4_·7H_2_O 8.85‍ ‍μM, EDTA·2Na 11.3‍ ‍μM, glucose 1.0‍ ‍g L^–1^, MgCl_2_ 20‍ ‍mM, sucrose 0.5 M, and maleic acid 20‍ ‍mM) modified slightly from NBMSM (nutrient broth with magnesium-sucrose-maleic acid) medium (Kawai *et al.*, 2015). These bacteria were cultured at 27°C with agitation at 150 rpm until the middle exponential phase, and cell shapes were investigated by epifluorescence microscopy (Leica, DMI4000).

### Epifluorescence microscopy observations

Insects were dissected in phosphate-buffered saline (PBS) buffer under a binocular (Leica, S8APO), and M4 crypts were collected. To observe *in vivo* bacterial cells, M4 crypts were transferred onto a glass microscopy slide with 5‍ ‍μL of PBS buffer and a cover glass. Bacteria were released from the crypts by exerting pressure on the cover glass. They were observed by epifluorescence microscopy. *In vitro* cultured bacterial cells were similarly observed.

### Confocal laser scanning microscopy observations

In spatiotemporal observations of M4-colonizing Δ*amiC* mutant cells, symbiotic insects at the early 3rd instar stage (a few hours after molting from the 2nd instar), 3rd instar middle stage (1~2 days after molting from the 2nd instar), and 3rd instar late stage (3~4 days after molting from the 2nd instar) were dissected and the posterior region of the midgut was collected. Tissue was stained by 3.75‍ ‍μg mL^–1^ 4,6′-diamidino-2-phenylindole (DAPI), washed with PBS, fixed by 1% paraformaldehyde, washed again with PBS, mounted in 20% glycerol, and then observed by confocal laser scanning microscopy using the Leica TCS SP8 instrument.

### Quantitative analysis of bacterial cell shapes

Bacterial cells were randomly selected from microscopy images and cell shapes were evaluated using ImageJ software (ver.1.51) (Schneider *et al.*, 2012). An individual cell was selected as a Region Of Interest (ROI) and the lengths of the minor axis and major axis were measured. The aspect ratio as an index of circularity was calculated by dividing the minor axis length by the major axis length. Cell shape was assessed by the aspect ratio shown in Fig. S1. Parameters were measured in dividing cells and chain-forming cells. The number of cells investigated under each condition is shown in each figure (Fig. 2, 4, and S3).

## Results

### The Δ*amiC* mutant of *B. insecticola* forms cell chains *in vitro* and swollen cells in the bean bug midgut

**Fig. 1.**
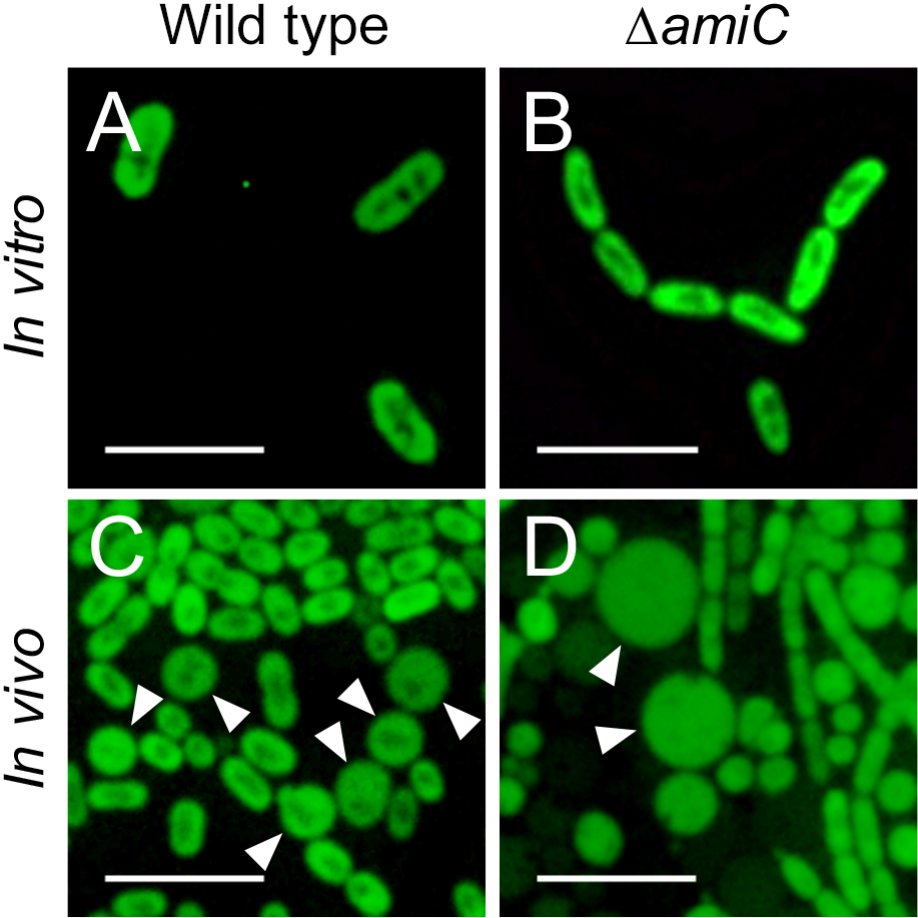
*In vitro* and *in vivo* cell morphologies of *Burkholderia insecticola* wild-type strain RPE225 and the Δ*amiC* mutant. Epifluorescence microscopy photos of (A, B) *in vitro* (cultured in YG medium) and (C, D) *in vivo* (colonizing M4 crypts at the 3rd instar nymph) bacterial cells in (A, C) wild-type strain RPE225 and (B, D) the Δ*amiC* mutant. Arrowheads indicate (C) spherical cells and (D) swollen cells. A quantitative analysis of each cell sample is shown in Fig. 2. Scale bar: 5‍ ‍μm.

The *B. insecticola* wild-type strain was rod-shaped when cultured *in vitro* (Fig. 1A), whereas *in vivo* symbiont cells became smaller and some were spherical, which is consistent with our previous findings (Fig. 1C; Ohbayashi *et al.*, 2019). In contrast, Δ*amiC* had a markedly different cell shape. The mutant formed long chains of unseparated cells in nutrient-rich YG medium due to incomplete cell division, as shown previously (Fig. 1B; Lee *et al.*, 2015), whereas *in vivo *Δ*amiC* cells became spherical and chain formation was less pronounced (Fig. 1D). Some spherical cells were very swollen (Fig. 1D).

**Fig. 2.**
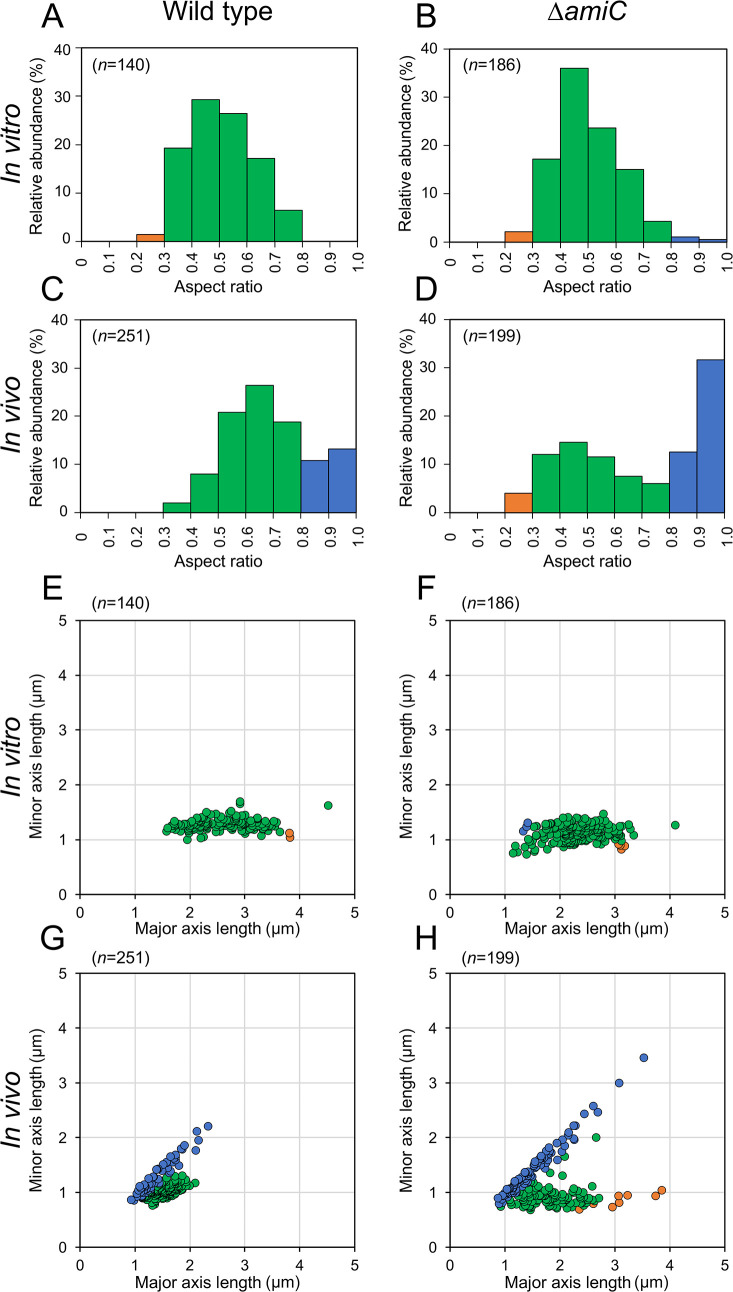
Relative abundance of cellular morphologies in the *in vitro* and *in vivo Burkholderia insecticola* wild type and Δ*amiC* mutant. Relative abundance of cellular morphology by (A–D) the aspect ratio distribution and by (E–H) the major axis and minor axis lengths distribution in the *in vitro* grown (A, E) wild type and (B, F) Δ*amiC* mutant and the *in vivo* (C, G) wild type and (D, H) Δ*amiC* mutant. Colors indicate bacterial cell morphologies. Orange indicates elongated cells with an aspect ratio less than 0.3, green indicates rod-shaped cells with an aspect ratio between 0.3 and 0.8, and blue indicates spherical cells with an aspect ratio between 0.8 and 1.0. The number of cells investigated under each condition is shown in each figure.

To quantitatively confirm morphological alterations in the Δ*amiC* mutant, an image analysis of cell shape was performed by measuring the minor axis and major axis lengths of individual cells in epifluorescence microscopy images. The aspect ratio, calculated as the division of the minor axis length by the major axis length, indicates the cellular circulation level, and the lengths of the major axis and minor axis were used as a proxy to estimate bacterial cell size (Fig. S1A). The aspect ratio varies between 0 and 1. A value close to 1 indicates a spherical cell shape, whereas strongly elongated cells have a value close to 0 (Fig. S1B). The aspect ratio distribution of the wild type and Δ*amiC* mutant grown *in vitro* were indistinguishable and had a normal distribution with a mean aspect ratio of 0.5 (Fig. 2A and B). However, the aspect ratio of the *in vivo* symbiont cells of both the wild type and mutant had a bi-modal distribution, which was different from the aspect ratios of cells grown *in vitro* (Fig. 2C and D). Wild-type symbiont cells had an aspect ratio peak at 0.6 to 0.7, which was slightly higher than that when grown in culture, and a second peak at 0.9 to 1.0. However, the mutant had a similar first peak to bacteria grown *in vitro* and a second major peak at 0.9 to 1.0. The proportion of spherical bacterial cells with an aspect ratio larger than 0.8 was 44% for the *in vivo *Δ*amiC* mutant, whereas it was only 24% for the *in vivo* wild type (Fig. 2C and D), showing that the alteration from the rod shape to a spherical form was more pronounced in the mutant than in the wild type.

The distribution of the major and minor axis lengths of the wild type and Δ*amiC* mutant reflected cell size reductions *in vivo*, as shown previously (Fig. 2E, F, G, and H; Ohbayashi *et al.*, 2019). The wild type had a mean major axis length of 2.6‍ ‍μm *in vitro* and only 1.6‍ ‍μm *in vivo*, whereas the mean major axis length of the Δ*amiC* mutant was 2.3‍ ‍μm *in vitro* and 1.8‍ ‍μm *in vivo* (Fig. 2E, F, G, and H). Moreover, cell size measurements revealed the presence of a small fraction of spherical *in vivo *Δ*amiC* mutant cells with axis lengths larger than 2.3‍ ‍μm and up to 3.5‍ ‍μm. These very enlarged swollen cells were absent in the wild type (Fig. 2E, F, G, and H). Collectively, these results confirmed microscopic observations (Fig. 1D) and suggested that *in vivo* bacterial cells had two populations with rod-shaped and spherical cells and that the majority of Δ*amiC* mutant *in vivo* cells became spherical or swollen, with some becoming very large. Despite this marked difference in cell morphology, an infection test and the quantification of occupancy by bacteria demonstrated that the Δ*amiC* mutant colonized M4 crypts, similar to the wild type, in all nymph stages (Fig. S2).

### Fosfomycin induces swollen cells *in vitro* in the *B. insecticola *Δ*amiC* mutant

**Fig. 3.**
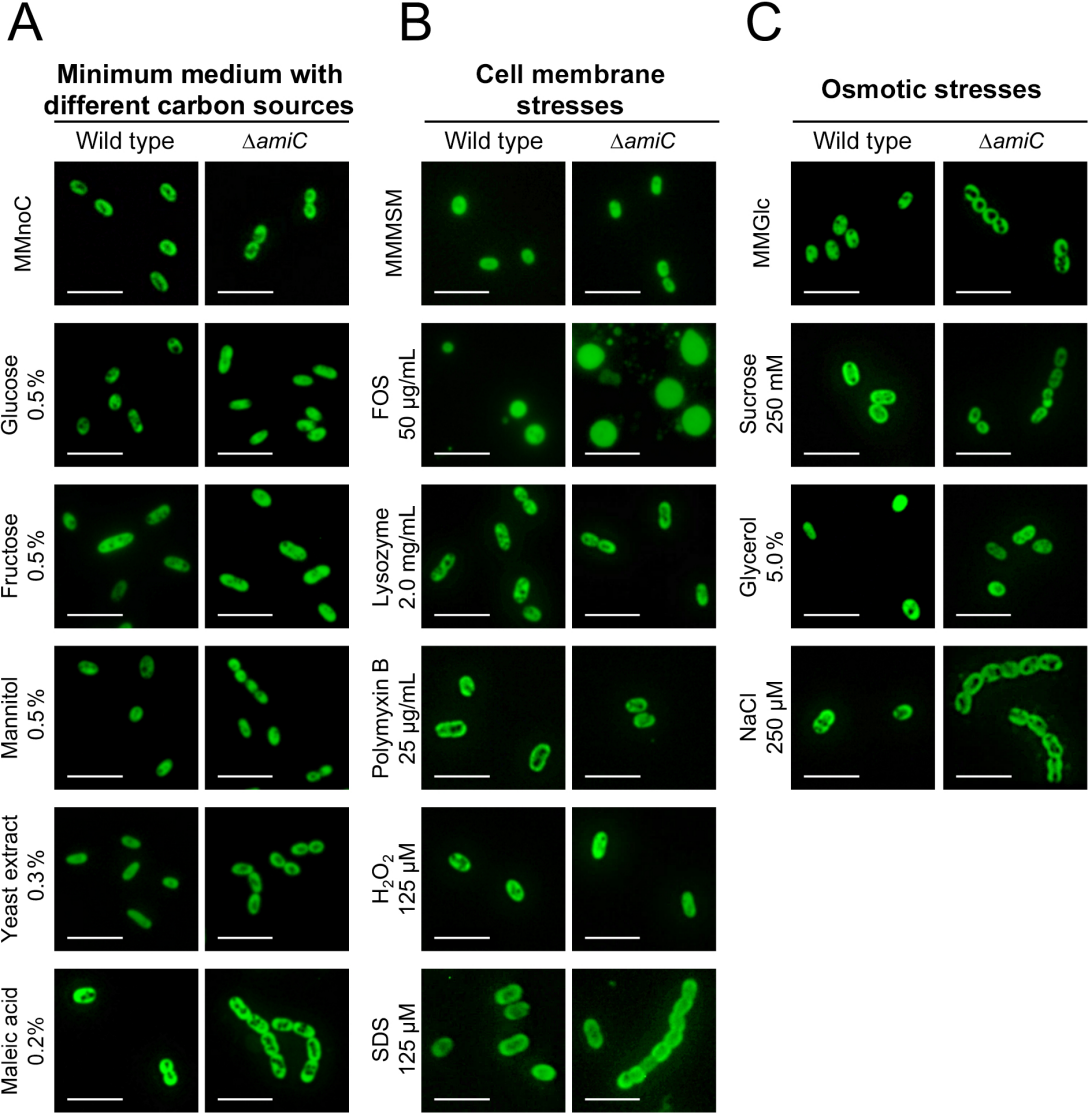
*In vitro* induction of swollen cells in the *Burkholderia insecticola* wild type and Δ*amiC* mutant. Epifluorescence microscopy images of wild-type and Δ*amiC* mutant cells cultured in (A) different carbon sources and after exposure to (B) cell membrane stresses or (C) osmotic stresses. Quantitative analyses of each cell sample are shown in Fig. 4 and S3. Scale bar: 5‍ ‍μm.

**Fig. 4.**
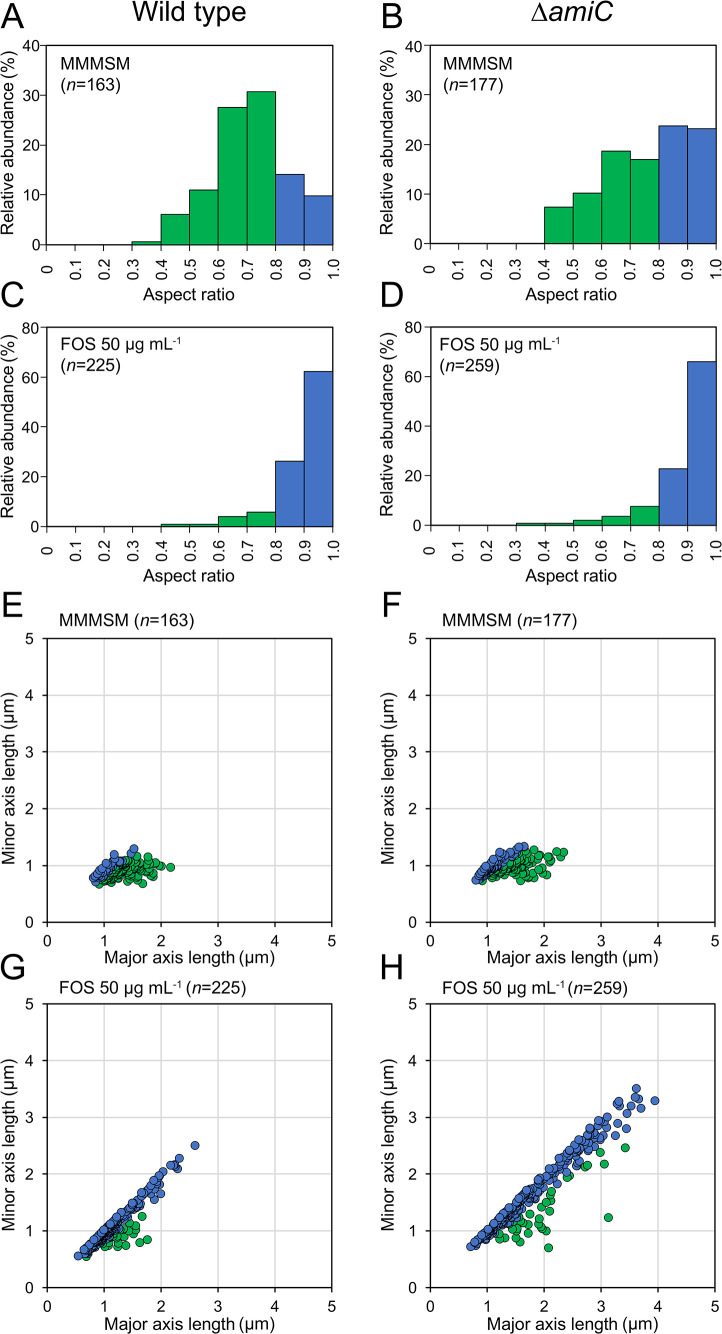
Distribution of cellular morphologies in the *Burkholderia insecticola* wild type and Δ*amiC* mutant after exposure to fosfomycin. Cellular morphology assessed by (A–D) the aspect ratio distribution and by (E–H) the major axis and minor axis lengths distribution in (A, B, E, F) osmoprotective MMMSM medium and (C, D, G, H) after exposure to fosfomycin in MMMSM medium in (A, C, E, G) the wild type and (B, D, F, H) Δ*amiC* mutant. The color code indicates bacterial cell morphologies, as in Fig. 2. The number of cells investigated in each condition is shown in each figure.

Many bacteria change their cell morphology based on environmental conditions, such as nutrient availability and bacterial stress (van Teeseling *et al.*, 2017). The mechanisms contributing to the production of swollen Δ*amiC* mutant cells in the midgut environment of the bean bug remain unclear. Therefore, we attempted to reproduce swollen cells *in vitro*. We investigated the cellular morphologies of the wild type and Δ*amiC* mutant cultured under 13 different conditions. These conditions included growth in minimal medium with five different carbon sources (glucose, fructose, mannitol, yeast extract, and maleic acid) and exposure to four cell membrane stresses (fosfomycin, lysozyme, polymyxin B, and SDS), oxidative stress (hydrogen peroxide), and three osmotic stresses (sucrose, glycerol, and NaCl). Among the 13 different culture conditions examined, only fosfomycin exposure induced spherical cells in the *B. insecticola* wild type and swollen cells in the Δ*amiC* mutant (Fig. 3). A quantitative analysis (Fig. 4) demonstrated that fosfomycin exposure resulted in the distribution of the cell shape aspect ratio with one sharp peak close at 0.9 to 1.0 in the wild type and Δ*amiC* mutant, similar to the major population of the *in vivo *Δ*amiC* mutant (Fig. 2D and 4C and D). Moreover, in the fosfomycin-exposed Δ*amiC* mutant, 25% of spherical cells had axis lengths of 2.3 to 3.9‍ ‍μm, similar to the largest swollen *in vivo* cells of the mutant (Fig. 2H and 4H). Fosfomycin is an inhibitor of the enzyme UDP-*N*-acetylglucosamine enolpyruvyl transferase (MurA), which is involved in the biosynthesis of the peptidoglycan precursor UDP-*N*-acetylglucosamine enolpyruvate (Falagas *et al.*, 2016), suggesting the induction of swollen Δ*amiC* mutant cells by the complete or partial lack of a cell wall. Consistent with this hypothesis, the observation of viable fosfomycin-treated Δ*amiC* mutant cells was only possible in the presence of 0.5 M osmoprotective sucrose, while the bacterium was uncultivable in non-osmoprotective minimal medium (data not shown).

### Swollen cells of the Δ*amiC* mutant are induced in the bean bug midgut prior to the molting stage

**Fig. 5.**
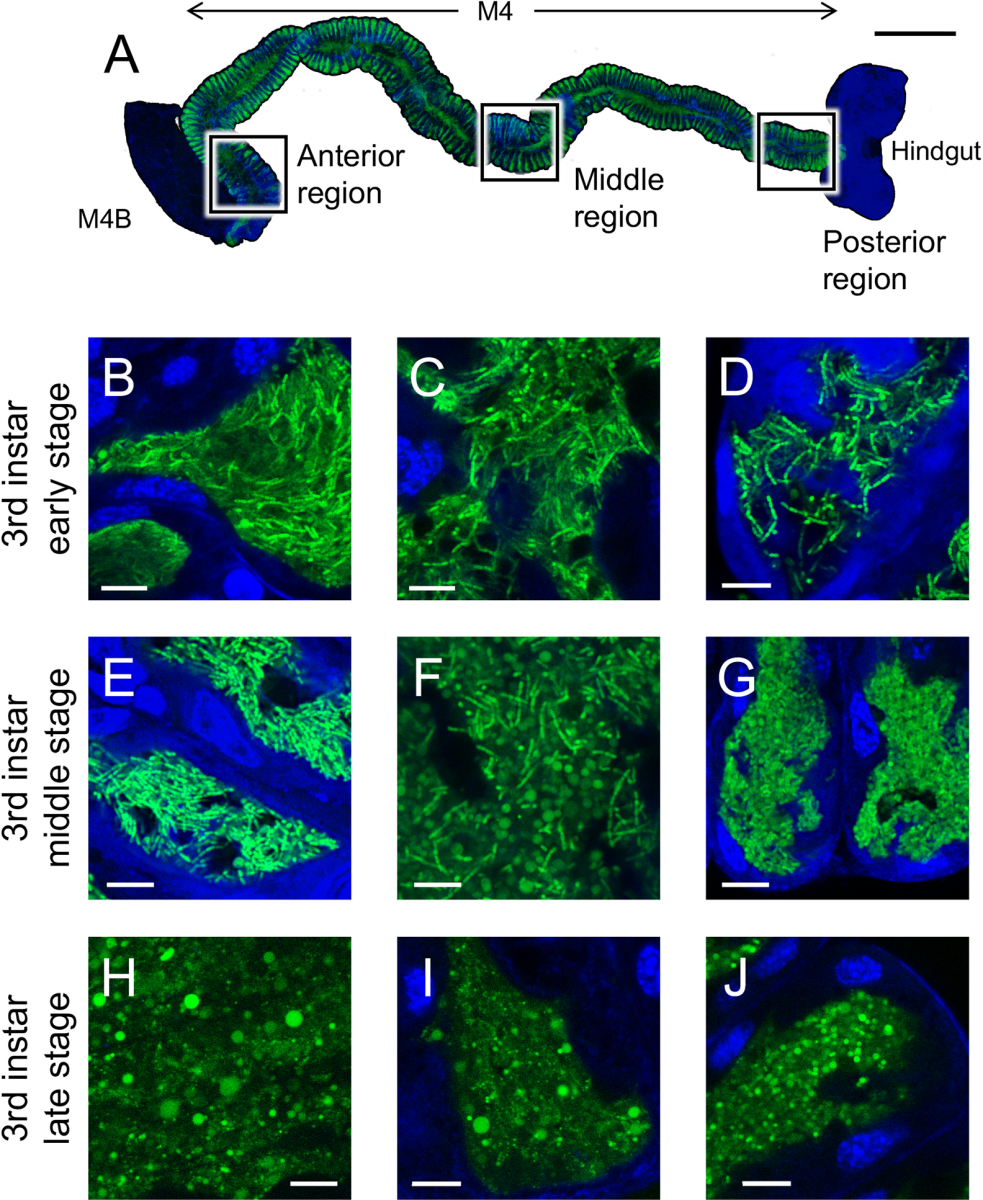
Cell morphology of the Δ*amiC* mutant colonizing M4 crypts. (A) The whole region of M4 crypts of a 3rd instar nymph of *Riptortus pedestris*. Scale bar: 0.5‍ ‍mm. (B–J) The Δ*amiC* mutant colonizing M4 crypts (B–D) at the 3rd instar early stage, (E–G) at the 3rd instar middle stage, and (H–J) at the 3rd instar late stage. (B, E, H) The anterior region, (C, F, I) middle region, and (D, G, J) posterior region of M4 crypts are shown. Green and blue signals indicate symbiont-derived GFP and host nuclear DNA, respectively. Scale bar: 10‍ ‍μm.

*B. insecticola* symbiont cells actively proliferate in M4 crypts with slight fluctuations in symbiont numbers during host development due to an increase in antimicrobial activity in M4 crypts before molting (Kim *et al.*, 2014; Ohbayashi *et al.*, 2019). We examined spatiotemporal variations in the *in vivo* cell shape of the Δ*amiC* mutant to establish whether host physiology affects symbiont morphology during host development (Fig. 5A). The Δ*amiC* mutant formed chains in the whole midgut region at the early 3rd instar stage (Fig. 5B, C, and D); however, many spherical cells were observed in the middle and posterior regions of M4 crypts at the middle 3rd instar stage (Fig. 5F and G). Most cells became spherical at all regions of M4 crypts at the late 3rd instar stage immediately prior to molting (Fig. 5H, I, and J), and some were very swollen in the anterior and middle regions (Fig. 5H, I, and J). These results suggested that host physiology before molting induced swollen cells in the *B. insecticola *Δ*amiC* mutant.

## Discussion

The present results revealed that the cell shape of the Δ*amiC* mutant of *B. insecticola* became swollen in the midgut crypts of the bean bug (Fig. 1D), and among the various *in vitro* conditions examined, only fosfomycin exposure induced a similar swollen shape in the Δ*amiC* mutant (Fig. 3). Moreover, the wild type showed a similar, but milder phenotype (Fig. 1C and D). These results indicate that the morphological alterations observed in the *Burkholderia* symbiont in the midgut crypts (Ohbayashi *et al.*, 2019) were induced by a cell wall-targeting agent.

The *in vitro* mimicking of swollen cells by fosfomycin strongly suggests that the swollen shape of the Δ*amiC* mutant was due to the complete or partial lack of a cell wall. Bacteria did not grow in non-osmoprotective minimal medium with fosfomycin. Bacterial lysis may have occurred due to impaired cell wall integrity, resulting from the inhibition of peptidoglycan precursor biosynthesis by fosfomycin combined with the deletion of the *amiC* gene. In the presence of the osmoprotectant sucrose, lysis was prevented in the fosfomycin-treated Δ*amiC* mutant and cells became spherical and enlarged. This morphology resembles the shape of cell wall-deficient bacterial forms, called the “L-form” (Allan *et al.*, 2009).

The “L-form” was initially described in *Streptobacillus moniliformis* (“L” was named in honor of the Lister Institute where the bacterial form was discovered) (Klieneberger, 1935). This pleomorphic bacterium switches its morphology from chains to swollen cells *in vitro* (Dienes, 1939). L-form bacteria were subsequently shown to be induced by the suppression of cell wall integrity with cell wall-inhibiting agents, such as penicillin and lysozyme (Madoff, 1986). Previous studies demonstrated that the L-form may be induced under osmoprotective conditions by lytic enzymes and/or inhibitors of cell wall biosynthesis, such as lysozyme, penicillin G, ampicillin, and fosfomycin, in diverse bacterial species, including Gram-positive and -negative bacteria (Allan *et al.*, 2009; Errington *et al.*, 2016). In the case of *B. insecticola*, only fosfomycin induced L-form-like swollen cells (Fig. 3). Fosfomycin targets MurA, which performs the first step in peptidoglycan synthesis (Egan *et al.*, 2020). Peptidoglycan formation is complex, consisting of many biosynthesis and regulatory steps (Table S1). It is thus‍ ‍possible that other molecules inhibiting steps downstream of MurA may cause similar phenotypes to fosfomycin. It currently remains unclear why other lytic enzymes and/or inhibitors of the cell wall, including lysozyme, polymyxin B, and SDS, did not induce the swollen cell shape (Fig. 3). We speculated that *Burkholderia* species have multiple defense mechanisms against surface-attacking antimicrobial agents, which may maintain the cell shape of the Δ*amiC* mutant under membrane stress conditions. For example, the modified LPS lipid A structure with positively charged 4-amino-4-deoxy-arabinose (Ara4N), membrane-integrated hopanoids, and several efflux pump mechanisms contribute to this cell envelope stability (Rhodes and Schweizer, 2016). C-type lysozymes, similar to that used in our assays, are known to hydrolyze the peptidoglycan of Gram-positive and -negative bacteria. However, since peptidoglycan in Gram-negative bacteria is shielded by the outer membrane, the enzyme may not have had access to the peptidoglycan substrate of *B. insecticola* in our assay. The symbiotic organ of *R. pedesteris* expresses genes encoding c-type lysozymes as well as a bacterial type lysozyme (Futahashi *et al.*, 2013). These enzymes may contribute to cell morphology modifications in the symbiotic organ, but may require the assistance of other factors in the gut that permeabilize the outer membrane. The absence of these factors may then explain the lack of a response by *B. insecticola* in our lysozyme assay. On the other hand, *B. insecticola* may also have a resistance mechanism against lysozyme activity as an adaptation for the colonization of lysozyme-containing midgut crypts.

Spatiotemporal observations of the Δ*amiC* mutant in midgut crypts revealed that symbiont cells became swollen, particularly before molting (Fig. 5). Kim *et al.* (2014) reported that a temporary decrease in the symbiont population occurred before molting, when the expression of genes encoding antimicrobial proteins and peptides, such as c-type lysozyme and riptocin, was up-regulated. In addition, these antimicrobial peptides produced in M4 crypts showed markedly stronger antimicrobial activity against *in vivo* symbiont cells than *in vitro* grown cells (Kim *et al.*, 2015; Ohbayashi *et al.*, 2019). The cell envelope structure of *B. insecticola* is altered *in vivo*, as revealed by the lack of the O-antigen polysaccharide on LPS and by the formation of blebs on the bacterial membrane (Kim *et al.*, 2015; Ohbayashi *et al.*, 2019). These stresses attacking the cell surface together with the deletion of the *amiC* gene may induce the swollen cells in M4 crypts before molting.

Although the present results suggest that a peptidoglycan-targeting antimicrobial agent is secreted in M4 crypts that induces the swollen cell shape, the nature of this agent remains unclear. In the case of legume-rhizobium symbiosis, NCR peptides in nodules block cell division in rhizobia, which induces cell elongation in bacteroids (Mergaert, 2018,‍ ‍^2020^). Strikingly, a rhizobium mutant in a gene encoding a peptidoglycan-modifying DD-carboxypeptidase enzyme forms bacteroids that have a strongly enlarged and spherical shape, similar to the crypt bacteria produced by the Δ*amiC* mutant described here. This mutant only forms these abnormally swollen bacteroids in nodules that produce NCR peptides, suggesting that these peptides are the direct cause of the bacterial growth defect of the mutant (Gully *et‍ ‍al.*, 2016). Bacteriocytes in the pea aphid produce bacteriocyte-specific cysteine-rich (BCR) peptides (Shigenobu and Stern, 2013). Although their *in vivo* functions currently remain unknown, some BCR peptides exhibited *in vitro* antimicrobial activity, induced cell elongation, and increased membrane permeability and DNA content in *E. coli*, which is phylogenetically close to *Buchnera* symbionts (Uchi *et al.*, 2019). These BCR peptides may affect the swollen and spherical morphology of *Buchnera* cells in bacteriocytes. In the bean bug, more than 90 species of crypt-specific cysteine-rich peptides (CCRs) are secreted in M4 crypts (Futahashi *et al.*, 2013), some of which exhibit antimicrobial activity *in vivo* against the cells of *B. insecticola* (Ohbayashi *et al.*, 2019). Although there is no sequence similarity between NCR, BCR, and CCR peptides, these peptides are all small and rich in cysteine, resembling the typical antimicrobial peptides of innate immunity, such as defensins (Mergaert *et al.*, 2003; Futahashi *et al.*, 2013; Shigenobu and Stern, 2013; Montiel *et al.*, 2017). This type of cysteine-rich peptide may be the result of convergent evolution in the different symbiotic systems in which they have similar functions. Future studies are needed to establish whether CCRs are involved in the morphological alterations of the Δ*amiC* mutant in midgut crypts by exposing the Δ*amiC* mutant to CCR peptides *in vitro* or by analyzing the morphology of the Δ*amiC* mutant in crypts after the suppression of CCR expression by reverse genetics in the host insect.

In conclusion, a detailed analysis of the symbiotic phenotype of the Δ*amiC* mutant enabled us to highlight the existence of a novel and partially characterized molecular mechanism of symbiont control by *R. pedestris* that may be widespread in host-microbe symbioses.

## Citation

Goto, S., Ohbayashi, T., Takeshita, K., Sone, T., Matsuura, Y., Mergaert, P., and Kikuchi, Y. (2020) A Peptidoglycan Amidase Mutant of *Burkholderia insecticola* Adapts an L-form-like Shape in the Gut Symbiotic Organ of the Bean Bug *Riptortus pedestris*. *Microbes Environ ***35**: ME20107.

https://doi.org/10.1264/jsme2.ME20107

## Supplementary Material

Supplementary Material

## References

[B1] Allan E.J., Hoischen C., and Gumpert J. (2009) Bacterial L‐forms. Adv Appl Microbiol 68: 1–39. 19426852 10.1016/S0065-2164(09)01201-5

[B2] Baumann P. (2005) Biology of bacteriocyte-associated endosymbionts of plant sap-sucking insects. Annu Rev Microbiol 59: 155–189. 16153167 10.1146/annurev.micro.59.030804.121041

[B3] Bublitz D.C., Chadwick G.L., Magyar J.S., Sandoz K.M., Brooks D.M., Mesnage S., et al. (2019) Peptidoglycan production by an insect-bacterial mosaic. Cell 179: 703–712. 31587897 10.1016/j.cell.2019.08.054 PMC6838666

[B4] Czernic P., Gully D., Cartieaux F., Moulin L., Guefrachi I., Patrel D., et al. (2015) Convergent evolution of endosymbiont differentiation in dalbergioid and inverted repeat-lacking clade legumes mediated by nodule-specific cysteine-rich peptides. Plant Physiol 169: 1254–1265. 26286718 10.1104/pp.15.00584 PMC4587450

[B5] Dienes L. (1939) L organisms of Klieneberger and *Streptobacillus moniliformis*. J Infect Dis 65: 24–42.

[B6] Egan A.J.F., Errington J., and Vollmer W. (2020) Regulation of peptidoglycan synthesis and remodelling. Nat Rev Microbiol 18: 446–460. 32424210 10.1038/s41579-020-0366-3

[B7] Errington J., Mickiewicz K., Kawai Y., and Wu L.J. (2016) L-form bacteria, chronic diseases and the origins of life. Philos Trans R Soc Lond B Biol Sci 371: 20150494. 27672147 10.1098/rstb.2015.0494 PMC5052740

[B8] Falagas M.E., Vouloumanou E.K., Samonis G., and Vardakas K.Z. (2016) Fosfomycin. Clin Microbiol Rev 29: 321–347. 26960938 10.1128/CMR.00068-15 PMC4786888

[B9] Futahashi R., Tanaka K., Tanahashi M., Nikoh N., Kikuchi Y., Lee B.L., et al. (2013) Gene expression in gut symbiotic organ of stinkbug affected by extracellular bacterial symbiont. PLoS One 8: e64557. 23691247 10.1371/journal.pone.0064557 PMC3653873

[B10] Gully D., Gargani D., Bonaldi K., Grangeteau C., Chaintreuil C., Fardoux J., et al. (2016) A peptidoglycan-remodeling enzyme is critical for bacteroid differentiation in *Bradyrhizobium* spp. during legume symbiosis. Mol Plant Microbe Interact 29: 447–457. 26959836 10.1094/MPMI-03-16-0052-R

[B11] Heidrich C., Templin M.F., Ursinus A., Merdanovic M., Berger J., Schwarz H., et al. (2001) Involvement of N‐acetylmuramyl‐L‐alanine amidases in cell separation and antibiotic‐induced autolysis of *Escherichia coli*. Mol Microbiol 41: 167–178. 11454209 10.1046/j.1365-2958.2001.02499.x

[B12] Hirota B., Okude G., Anbutsu H., Futahashi R., Moriyama M., Meng X.Y., et al. (2017) A novel, extremely elongated, and endocellular bacterial symbiont supports cuticle formation of a grain pest beetle. mBio 8: e01482-17. 28951480 10.1128/mBio.01482-17 PMC5615201

[B13] Itoh H., Jang S., Takeshita K., Ohbayashi T., Ohnishi N., Meng X.Y., et al. (2019) Host-symbiont specificity determined by microbe-microbe competition in an insect gut. Proc Natl Acad Sci U S A 116: 22673–22682. 31636183 10.1073/pnas.1912397116 PMC6842582

[B14] Kaltenpoth M., and Flórez L.V. (2020) Versatile and dynamic symbioses between insects and *Burkholderia* bacteria. Annu Rev Entomol 65: 145–170. 31594411 10.1146/annurev-ento-011019-025025

[B15] Kawai Y., Mercier R., Wu L.J., Domínguez-Cuevas P., Oshima T., and Errington J. (2015) Cell growth of wall-free L-form bacteria is limited by oxidative damage. Curr Biol 25: 1613–1618. 26051891 10.1016/j.cub.2015.04.031 PMC4510147

[B16] Kikuchi Y., Hosokawa T., and Fukatsu T. (2007) Insect-microbe mutualism without vertical transmission: a stinkbug acquires a beneficial gut symbiont from the environment every generation. Appl Environ Microbiol 73: 4308–4316. 17483286 10.1128/AEM.00067-07 PMC1932760

[B17] Kikuchi Y., Hosokawa T., and Fukatsu T. (2011) Specific developmental window for establishment of an insect-microbe gut symbiosis. Appl Environ Microbiol 77: 4075–4081. 21531836 10.1128/AEM.00358-11 PMC3131632

[B18] Kikuchi Y., and Fukatsu T. (2014) Live imaging of symbiosis: spatiotemporal infection dynamics of a GFP-labelled *Burkholderia* symbiont in the bean bug *Riptortus pedestris*. Mol Ecol 23: 1445–1456. 24103110 10.1111/mec.12479 PMC4238818

[B19] Kim J.K., Han S.H., Kim C.H., Jo Y.H., Futahashi R., Kikuchi Y., et al. (2014) Molting-associated suppression of symbiont population and up-regulation of antimicrobial activity in the midgut symbiotic organ of the *Riptortus-Burkholderia* symbiosis. Dev Comp Immunol 43: 10–14. 24201132 10.1016/j.dci.2013.10.010

[B20] Kim J.K., Son D.W., Kim C.H., Cho J.H., Marchetti R., Silipo A., et al. (2015) Insect gut symbiont susceptibility to host antimicrobial peptides caused by alteration of the bacterial cell envelope. J Biol Chem 290: 21042–21053. 26116716 10.1074/jbc.M115.651158 PMC4543662

[B21] Klieneberger E. (1935) The natural occurrence of pleuropneumonia-like organisms in apparent symbiosis with *Streptobacillus moniliformis* and other bacteria. J Pathol Bacteriol 40: 93–105.

[B22] Lee J.B., Byeon J.H., Jang H.A., Kim J.K., Yoo J.W., Kikuchi Y., et al. (2015) Bacterial cell motility of *Burkholderia* gut symbiont is required to colonize the insect gut. FEBS Lett 589: 2784–2790. 26318755 10.1016/j.febslet.2015.08.022

[B23] Login F.H., Balmand S., Vallier A., Vincent-Monegat C., Vigneron A., Weiss-Gayet M., et al. (2011) Antimicrobial peptides keep insect endosymbionts under control. Science 334: 362–365. 22021855 10.1126/science.1209728

[B24] Lukasik, P., Newton, J.A., Sanders, J.G., Hu, Y., Moreau, C.S., Kronauer, D.J.C., *et al.* (2017) The structured diversity of specialized gut symbionts of the new world army ants. *Mol Ecol* **26**: 3808–3825. 10.1111/mec.14140 28393425

[B25] Madoff, S. (1986). *The Bacterial L-Forms*. New York, NY: Marcel Dekker.

[B26] Mergaert P., Nikovics K., Kelemen Z., Maunoury N., Vaubert D., Kondorosi A., et al. (2003) A novel family in *Medicago truncatula* consisting of more than 300 nodule-specific genes coding for small, secreted polypeptides with conserved cysteine motifs. Plant Physiol 132: 161–173. 12746522 10.1104/pp.102.018192 PMC166962

[B27] Mergaert P., Uchiumi T., Alunni B., Evanno G., Cheron A., Catrice O., et al. (2006) Eukaryotic control on bacterial cell cycle and differentiation in the *Rhizobium*-legume symbiosis. Proc Natl Acad Sci U S A 103: 5230–5235. 16547129 10.1073/pnas.0600912103 PMC1458823

[B28] Mergaert P. (2018) Role of antimicrobial peptides in controlling symbiotic bacterial populations. Nat Prod Rep 35: 336–356. 29393944 10.1039/c7np00056a

[B29] Mergaert P. (2020) Differentiation of symbiotic nodule cells and their rhizobium endosymbionts. Adv Bot Res 94: 149–180.

[B30] Montiel J., Downie J.A., Farkas A., Bihari P., Herczeg R., Balint B., et al. (2017) Morphotype of bacteroids in different legumes correlates with the number and type of symbiotic NCR peptides. Proc Natl Acad Sci U S A 114: 5041–5046. 28438996 10.1073/pnas.1704217114 PMC5441718

[B31] Ohbayashi T., Takeshita K., Kitagawa W., Nikoh N., Koga R., Meng X.Y., et al. (2015) Insect’s intestinal organ for symbiont sorting. Proc Natl Acad Sci U S A 112: E5179–E5188. 26324935 10.1073/pnas.1511454112 PMC4577176

[B32] Ohbayashi T., Futahashi R., Terashima M., Barriere Q., Lamouche F., Takeshita K., et al. (2019) Comparative cytology, physiology and transcriptomics of *Burkholderia insecticola* in symbiosis with the bean bug *Riptortus pedestris* and in culture. ISME J 13: 1469–1483. 30742016 10.1038/s41396-019-0361-8 PMC6776119

[B33] Ohbayashi T., Mergaert P., and Kikuchi Y. (2020) Host-symbiont specificity in insects: Underpinning mechanisms and evolution. Adv Insect Physiol 58: 27–62.

[B34] Oke V., and Long S.R. (1999) Bacteroid formation in the *Rhizobium*-legume symbiosis. Curr Opin Microbiol 2: 641–646. 10607628 10.1016/s1369-5274(99)00035-1

[B35] Okude G., Koga R., Hayashi T., Nishide Y., Meng X.Y., Nikoh N., et al. (2017) Novel bacteriocyte-associated pleomorphic symbiont of the grain pest beetle *Rhyzopertha dominica* (Coleoptera: Bostrichidae). Zoological Lett 3: 13. 28828177 10.1186/s40851-017-0073-8 PMC5563036

[B36] Rhodes K.A., and Schweizer H.P. (2016) Antibiotic resistance in *Burkholderia* species. Drug Resist Updates 28: 82–90. 10.1016/j.drup.2016.07.003 PMC5022785 27620956

[B37] Schmidtke L.M., and Carson J. (1999) Induction, characterisation and pathogenicity in rainbow trout *Oncorhynchus mykiss* (Walbaum) of *Lactococcus garvieae* L-forms. Vet Microbiol 69: 287–300. 10535774 10.1016/s0378-1135(99)00120-0

[B38] Schneider C.A., Rasband W.S., and Eliceiri K.W. (2012) NIH Image to ImageJ: 25 years of image analysis. Nat Methods 9: 671–675. 22930834 10.1038/nmeth.2089 PMC5554542

[B39] Shigenobu S., and Wilson A.C. (2011) Genomic revelations of a mutualism: the pea aphid and its obligate bacterial symbiont. Cell Mol Life Sci 68: 1297–1309. 21390549 10.1007/s00018-011-0645-2 PMC3064905

[B40] Shigenobu S., and Stern D.L. (2013) Aphids evolved novel secreted proteins for symbiosis with bacterial endosymbiont. Philos Trans R Soc Lond B Biol Sci 280: 20121952. 10.1098/rspb.2012.1952 PMC3574423 23173201

[B41] Strang J.A., Allan E.J., Seddon B., and Paton A.M. (1991) Induction of *Bacillus brevis* L-forms. J Appl Bacteriol 70: 47–51.

[B42] Takeshita K., and Kikuchi Y. (2017) *Riptortus pedestris* and *Burkholderia* symbiont: an ideal model system for insect-microbe symbiotic associations. Res Microbiol 168: 175–187. 27965151 10.1016/j.resmic.2016.11.005

[B43] Uchi N., Fukudome M., Nozaki N., Suzuki M., Osuki K.I., and Shigenobu S., et al. (2019) Antimicrobial activities of cysteine-rich peptides specific to bacteriocytes of the pea aphid *Acyrthosiphon pisum*. Microbes Environ 34: 155–160. 30905896 10.1264/jsme2.ME18148 PMC6594739

[B44] van Teeseling M.C.F., de Pedro M.A., and Cava F. (2017) Determinants of bacterial morphology: from fundamentals to possibilities for antimicrobial targeting. Front Microbiol 8: 1264. 28740487 10.3389/fmicb.2017.01264 PMC5502672

[B45] Young K.D. (2006) The selective value of bacterial shape. Microbiol Mol Biol Rev 70: 660–703. 16959965 10.1128/MMBR.00001-06 PMC1594593

