# Supplementary material for: A Peptidoglycan Amidase Mutant of Burkholderia insecticola Adapts an L-form-like Shape in the Gut Symbiotic Organ of the Bean Bug Riptortus pedestris
Source: Microbes Environ. 2020 Nov 11;35(4):ME20107. doi: 10.1264/jsme2.ME20107 (PMC7734397; doi:10.1264/jsme2.ME20107)
Supplement: Supplementary file 1 — Supplementary Material [file 35_20107_s1.pdf]

Fig. S1

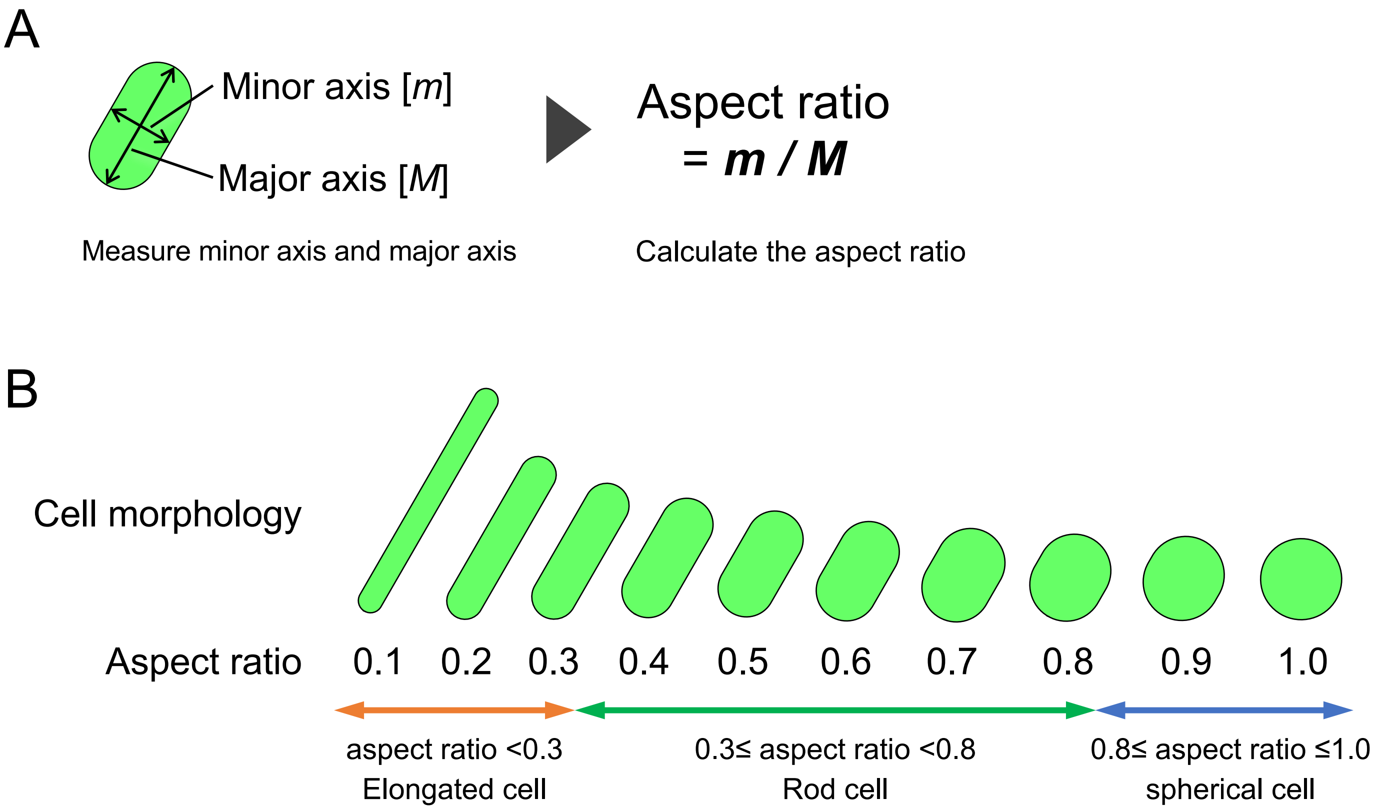

Supplementary Fig. S1. Illustration of the image analysis of cell shape

(A) The illustration of major and minor axis and aspect ratio in image analysis of cell shape. (B) The correspondence of aspect ratio to cell morphology. Aspect ratios less than 0.3 correspond to elongated cells (orange), aspect ratios between 0.3 and 0.8 correspond to rod-shaped cells (green), and aspect ratios between 0.8 and 1.0 correspond to spherical cells (blue). These color code is used in aspect ratio distribution and in major axis and minor axis lengths distribution to distinguish bacterial morphology of *B. insecticola* wild type and the Δ*amiC* mutant as in Fig. 2, Fig. 4 and Fig. S3.

Fig. S2

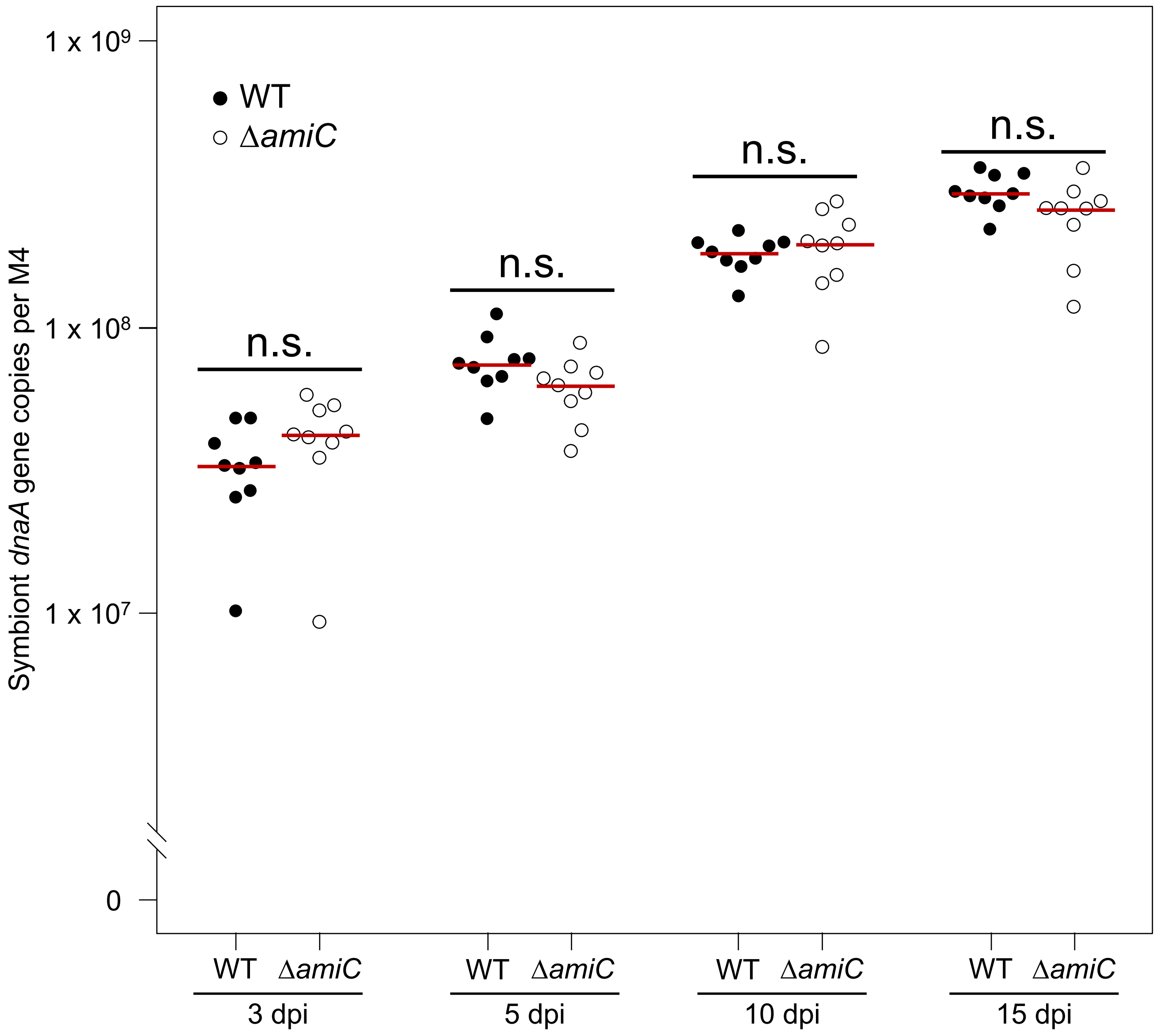

Supplementary Fig. S2. Colonization ability of the M4 crypts of *R. pedestris* in *B. insecticola* wild type and Δ*amiC* mutant

Quantitative PCR analysis of symbiont density of the M4 infected with *B. insecticola* wild type (closed circles) and Δ*amiC* mutant (open circles) at 3 dpi, 5 dpi, 10 dpi and 15 dpi. Nine insects were investigated in each condition. Red bars indicate the mean for each condition. The statistical difference between the wild type and Δ*amiC* mutant was investigated by *Weltch's t*-test for samples of 3 dpi, 5 dpi and 15 dpi, and by *Student's t*-test for 10 dpi (n.s.: not significant).

Fig. S3

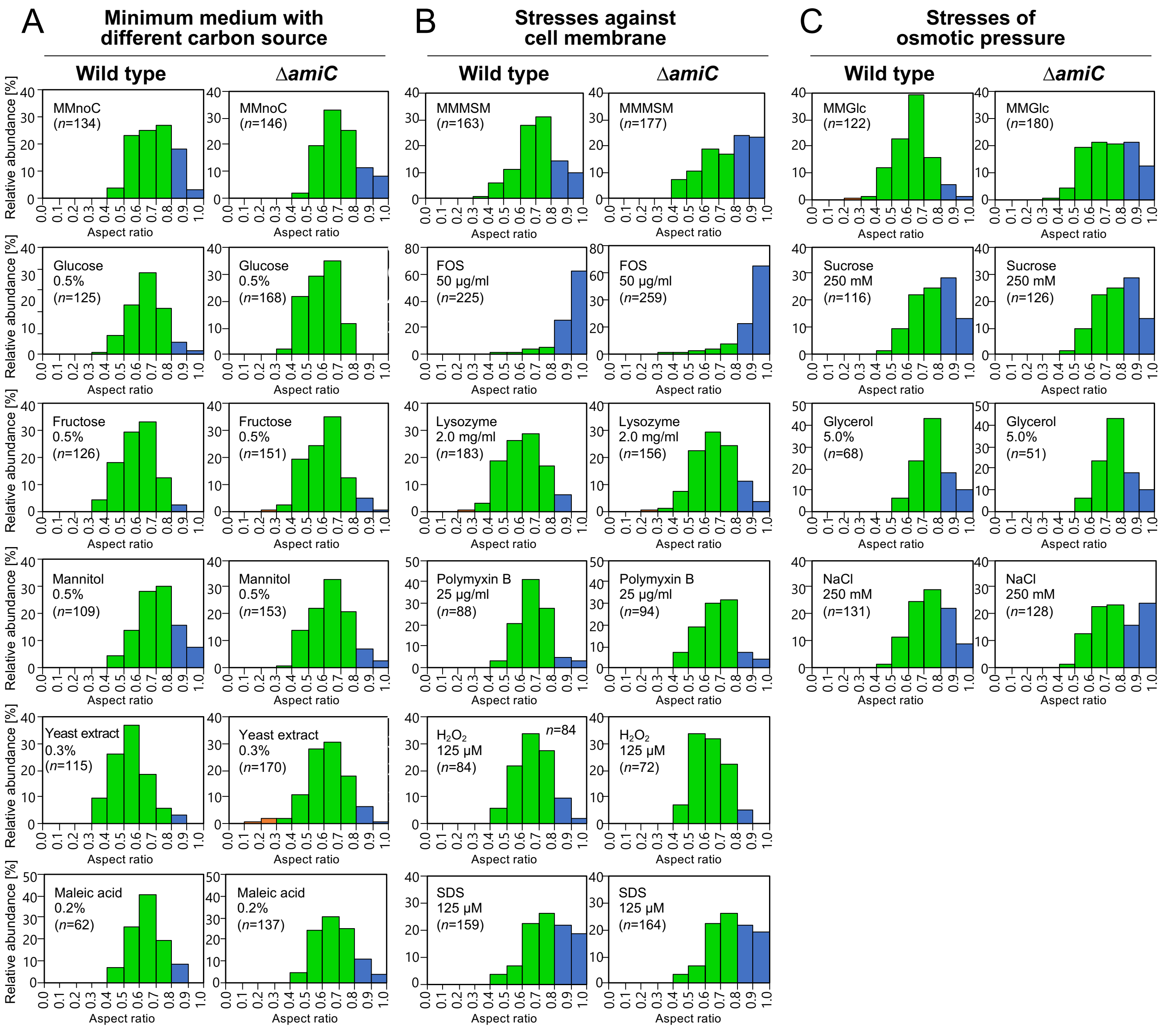

Supplementary Fig. S3. Distribution of cellular morphology in *in vitro B. insecticola* wild type and Δ*amiC* mutant

Cellular morphology by (A-C) aspect ratio distribution and by (D-F) major axis and minor axis lengths distribution in *B. insecticola* wild type and the Δ*amiC* mutant, cultured in (A, D) different carbon sources, or in the presence of (B, E) stresses against the cell membrane and (C, F) stresses of osmotic pressure. The color code indicates bacterial cell morphology as in Fig. 2. The number of investigated cells in each condition is shown in each panel.

Fig. S3-continued

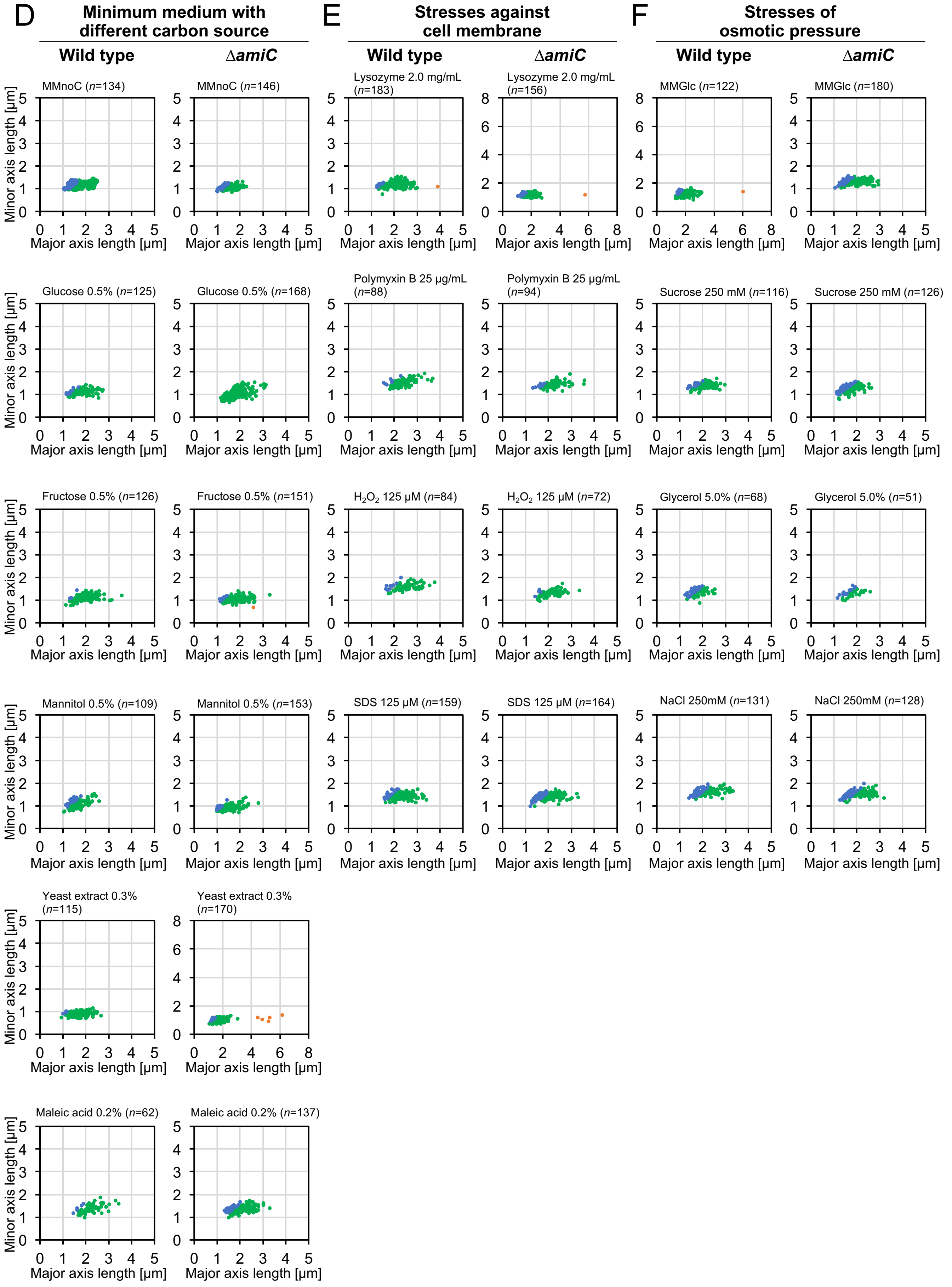

Table S1. List of peptidoglycan-targeting/recycling genes found in *R. pedestris* and *B. insecticola*

Accession number (EST-ID)	Gene name	Annotation	Source
AK416888.1 (Rped-0025)		c-type lysozyme	The midgut of *R. pedestris*
AK416923.1 (Rped-0069)		c-type lysozyme	The midgut of *R. pedestris*
AK416891.1 (Rped-0028)		bacterial type lysozyme	The midgut of *R. pedestris*
BRPE64_ACDS22630	*amiC*	N-acetylmuramoyl-L-alanine amidase	*B. insecticola*
BRPE64_ACDS16480	*amiC*	N-acetylmuramoyl-L-alanine amidase	*B. insecticola*
BRPE64_ACDS23830	*ampD*	N-acetylmuramyl-L-alanine amidase negative regulator of AmpC AmpD	*B. insecticola*
BRPE64_ACDS23830	*ampD*	N-acetyl-anhydromuramoyl-L-alanine amidase	*B. insecticola*
BRPE64_ACDS27170	*flgJ*	flagellar rod assembly protein/muramidase	*B. insecticola*
BRPE64_ACDS24380	*mltA*	membrane-bound lytic murein transglycosylase A	*B. insecticola*
BRPE64_ACDS20570	*mltB*	membrane-bound lytic murein transglycosylase B	*B. insecticola*
BRPE64_ACDS19400	*mltD*	membrane-bound lytic murein transglycosylase D	*B. insecticola*
BRPE64_ACDS26650	*slt*	lytic transglycosylase	*B. insecticola*
BRPE64_CCDS00320	*rlpA*	Septal ring protein, transglycolytic activity	*B. insecticola*
BRPE64_CCDS01540	*rlpA*	Septal ring protein, transglycolytic activity	*B. insecticola*
BRPE64_ACDS28740	*rlpA*	Septal ring protein, transglycolytic activity	*B. insecticola*
BRPE64_DCDS10670	*rlpA*	Septal ring protein, transglycolytic activity	*B. insecticola*
BRPE64_ACDS12560	*mrcA*	putative penicillin-binding (Peptidoglycan synthetase) transmembrane protein	*B. insecticola*
BRPE64_ACDS25430	*mrcA*	penicillin-binding protein 1A family	*B. insecticola*
BRPE64_CCDS08320	*mrcA*	peptidoglycan glycosyltransferase	*B. insecticola*
BRPE64_ACDS16160	*pbpC*	penicillin-binding protein	*B. insecticola*
BRPE64_ACDS00420	*mrdA*	penicillin-binding protein	*B. insecticola*
BRPE64_ACDS24230	*ftsI*	peptidoglycan glycosyltransferase	*B. insecticola*
BRPE64_BCDS05920	*ftsI*	peptidoglycan glycosyltransferase	*B. insecticola*
BRPE64_ACDS01710	*dacC*	penicillin-binding protein 6 Serine peptidase MEROPS family S11	*B. insecticola*
BRPE64_ACDS05000	*dacB*	D-alanyl-D-alanine carboxypeptidase/D-alanyl-D-alanine-endopeptidase	*B. insecticola*
BRPE64_ACDS00520	*ampG*	major facilitator superfamily MFS_1; Putative muropeptide permease	*B. insecticola*
BRPE64_BCDS08670	*oppA*	probable oligopeptide ABC transporter system substrate-binding protein	*B. insecticola*
BRPE64_BCDS08660	*oppB*	oligopeptide transport system permease protein	*B. insecticola*
BRPE64_BCDS08650	*oppC*	oligopeptide transport system permease protein	*B. insecticola*
BRPE64_CCDS06190	*oppD*	putative peptide ABC transporter ATP-binding protein	*B. insecticola*
BRPE64_BCDS08640	*oppD*	oligopeptide transport system ATP-binding protein	*B. insecticola*
BRPE64_CCDS01780	*oppD*	oligopeptide/dipeptide ABC transporter ATPase subunit	*B. insecticola*
BRPE64_CCDS06180	*oppF*	putative ABC transporter ATP-binding protein	*B. insecticola*
BRPE64_BCDS08630	*oppF*	oligopeptide ABC transport system ATP-binding protein	*B. insecticola*
BRPE64_ACDS02260	*nagE*	beta-N-acetylhexosaminidase	*B. insecticola*
BRPE64_ACDS12280	*ldcA*	murein tetrapeptidase LD-carboxypeptidase	*B. insecticola*
BRPE64_ACDS03320	*anmK*	anhydro-N-acetylmuramic acid kinase	*B. insecticola*
BRPE64_ACDS23080	*amgK*	putative N-acetylmuramate 1-kinase	*B. insecticola*
BRPE64_ACDS20770	*mupP*	putative N-acetyl-D-muramate 6-phosphate phosphatase	*B. insecticola*
BRPE64_ACDS23090	*murU*	nucleotidyl transferase family protein	*B. insecticola*
BRPE64_ACDS02230	*nagA*	N-acetylglucosamine-6-phosphate deacetylase	*B. insecticola*
BRPE64_ACDS23630	*mpl*	UDP-N-acetylmuramate	*B. insecticola*
